# Enhancing Green Fraction Estimation in Rice and Wheat Crops: A Self-Supervised Deep Learning Semantic Segmentation Approach

**DOI:** 10.34133/plantphenomics.0064

**Published:** 2023-07-18

**Authors:** Yangmingrui Gao, Yinglun Li, Ruibo Jiang, Xiaohai Zhan, Hao Lu, Wei Guo, Wanneng Yang, Yanfeng Ding, Shouyang Liu

**Affiliations:** ^1^ Plant Phenomics Research Centre, Academy for Advanced Interdisciplinary Studies, Jiangsu Collaborative Innovation Center for Modern Crop Production, Nanjing Agricultural University, Nanjing, China.; ^2^ Key Laboratory of Image Processing and Intelligent Control, School of Artificial Intelligence and Automation, Huazhong University of Science and Technology, Wuhan, China.; ^3^ Graduate School of Agricultural and Life Sciences, The University of Tokyo, 1-1-1 Midori-cho, Nishitokyo City, Tokyo, Japan.; ^4^ National Key Laboratory of Crop Genetic Improvement, National Center of Plant Gene Research, and Hubei Key Laboratory of Agricultural Bioinformatics, Huazhong Agricultural University, Wuhan 430070, China.

## Abstract

The green fraction (GF), which is the fraction of green vegetation in a given viewing direction, is closely related to the light interception ability of the crop canopy. Monitoring the dynamics of GF is therefore of great interest for breeders to identify genotypes with high radiation use efficiency. The accuracy of GF estimation depends heavily on the quality of the segmentation dataset and the accuracy of the image segmentation method. To enhance segmentation accuracy while reducing annotation costs, we developed a self-supervised strategy for deep learning semantic segmentation of rice and wheat field images with very contrasting field backgrounds. First, the Digital Plant Phenotyping Platform was used to generate large, perfectly labeled simulated field images for wheat and rice crops, considering diverse canopy structures and a wide range of environmental conditions (sim dataset). We then used the domain adaptation model cycle-consistent generative adversarial network (CycleGAN) to bridge the reality gap between the simulated and real images (real dataset), producing simulation-to-reality images (sim2real dataset). Finally, 3 different semantic segmentation models (U-Net, DeepLabV3+, and SegFormer) were trained using 3 datasets (real, sim, and sim2real datasets). The performance of the 9 training strategies was assessed using real images captured from various sites. The results showed that SegFormer trained using the sim2real dataset achieved the best segmentation performance for both rice and wheat crops (rice: Accuracy = 0.940, F1-score = 0.937; wheat: Accuracy = 0.952, F1-score = 0.935). Likewise, favorable GF estimation results were obtained using the above strategy (rice: *R*^2^ = 0.967, RMSE = 0.048; wheat: *R*^2^ = 0.984, RMSE = 0.028). Compared with SegFormer trained using a real dataset, the optimal strategy demonstrated greater superiority for wheat images than for rice images. This discrepancy can be partially attributed to the differences in the backgrounds of the rice and wheat fields. The uncertainty analysis indicated that our strategy could be disrupted by the inhomogeneity of pixel brightness and the presence of senescent elements in the images. In summary, our self-supervised strategy addresses the issues of high cost and uncertain annotation accuracy during dataset creation, ultimately enhancing GF estimation accuracy for rice and wheat field images. The best weights we trained in wheat and rice are available: https://github.com/PheniX-Lab/sim2real-seg.

## Introduction

Green fraction (GF) is a phenotypic trait associated with the photosynthetic capacity of crops. It is defined as the fraction of green vegetation in a given viewing direction [1]. The GF in the solar angle equals the fraction of light intercepted by green elements that drive the photosynthesis and transpiration processes [2,3]. In addition, GF can be used to estimate the green area index and characterize the canopy structure [4]. Monitoring the dynamics of GF is thus of great interest for breeders to quantitatively describe the process of crop growth and development, and consequently, guide their selection of cultivars adapted to the targeted environment [5].

The GF can be estimated from red-green-blue (RGB) images of the crop canopy by calculating the fraction of green pixels belonging to the crop [6]. The GF estimation accuracy relies on the performance of the segmentation algorithm, which discriminates between pixels of the green crops and others. This task is characterized by semantic segmentation in computer vision (CV). Existing algorithms primarily include traditional image processing and deep learning techniques. First, these traditional methods only use local color features to segment pixels with static thresholding [7–9] or shallow machine learning techniques [10,11]. This limits the robustness of these algorithms and makes them prone to variations in canopy structure and environmental conditions, particularly the fluctuation of outdoor illumination [12]. In contrast, deep learning techniques can extract more representative features on a larger scale. This explains to large extent the success of deep learning models in image segmentation [13–16].

Deep learning algorithms have been used for the segmentation of agricultural images, and the recently published SegVeg model has achieved much better performance than conventional methods [17]. Even so, SegVeg did not use the state-of-the-art (SOTA) semantic segmentation model based on the vision transformer, which has been proven on other CV datasets for better accuracy [18]. Generally, algorithms transferred into plant phenotyping lag behind SOTA algorithms developed in the CV community. This is because, in the CV community, the publicly available datasets greatly facilitate the deployment and development of algorithms. In contrast, for plant phenotyping, this type of dataset rarely exists [19]. Applying deep learning techniques to plant phenotyping often requires important effort to build a dataset [20,21]. Specifically, it is difficult to collect a large number of images that represent a wide range of variations, including genotypes, soil background, weather conditions, and sensor properties. Moreover, it is extremely laborious and tedious to annotate agricultural images manually, especially for semantic segmentation. Compared with the CV community dataset, more plant edges need to be labeled in the phenotypic dataset. These edges are often difficult to label because of shadows and low-resolution blurring. High labor costs and potential labeling errors hinder the application of the SOTA semantic segmentation algorithm in GF estimation.

Using a 3-dimensional (3D) model to generate synthetic images provides an alternative method for establishing a training dataset for deep learning [22–24]. The Digital Plant Phenotyping Platform (D3P) was developed to simulate phenotyping observations [4,25]. Owing to the D3P, simulated crop growth images and the corresponding binary labels can be generated automatically. Although we can generate simulated field rice and wheat semantic segmentation datasets at high throughput, the simulated images are still not sufficiently realistic for leaf texture and light distribution compared with real images captured in the field. This results in the feature distribution of the simulated and real images not being in the same domain. There is a large domain gap between the 2 datasets. Traditional image augmentation methods such as flipping and scaling involve only vector manipulations of the image, which cannot narrow the domain gap. Hence, style transfer based on domain adaptation techniques such as generative adversarial networks (GAN) [26] should be applied to enhance image realism and reduce the domain gap [27,28]. Our recent study was successful in wheat leaf tip detection by combining simulated images with domain adaptation techniques [29]. This pipeline is worth attempting for self-supervised semantic segmentation.

The main objective of this study was to extend the self-supervised plant phenotyping pipeline to build a semantic segmentation algorithm using RGB images. Because the field background remarkably affects the performance of segmentation algorithms, self-supervised segmentation algorithms can be evaluated on both rice and wheat crops with very contrasting field backgrounds. First, we collected in situ real images and their manual annotation labels from different sites (real dataset) and used D3P to automatically generate simulated images with accompanying labels (sim dataset). Second, the domain adaptation method cycle-consistent generative adversarial network (CycleGAN) [30] was used to minimize the domain gap between the sim dataset and the real dataset to establish the simulation-to-reality dataset (sim2real dataset). Finally, 3 representative deep learning models (U-Net, DeepLabV3+, and SegFormer) were selected, and their performances trained over 3 datasets (real, sim, and sim2real) were compared with the test dataset at the pixel scale and image scale (GF estimation). Furthermore, the factors affecting the estimation uncertainty were carefully inspected.

## Materials and Methods

### Field image collection

Ground-based RGB images of wheat and rice crops from several countries were captured and collected for semantic segmentation (Table 1). The dataset from Switzerland, Zurich, was obtained from a previously published article [31]. These images represent a wide range of canopy structural variations, considering the impacts of genotypes, field cultivation practices, and environmental conditions. Samples were collected at different phenological stages during the growth cycle. We also considered the impact of illumination conditions on images taken on sunny and cloudy days. All images were captured using high-definition sensors such as single-lens reflex cameras, portable action cameras, or smartphones. The height of the sensors was almost less than 2 m above the canopy, with a spatial resolution varying from 0.3 to 0.6 mm/pixel. The viewing angle was between 0^o^ looking downward and 45^o^ inclined toward the canopy. The original images were cropped around the center to the size of 512 × 512. Then, all real images were manually segmented using a JavaScript image annotation tool based on the JetBrains WebStorm software [32] (https://github.com/kyamagu/js-segment-annotator) to create the dataset for model training and testing, hereafter referred to as the real dataset. In the annotation process, all pixels were classified into 2 categories: green vegetation (including green pixels from all the organs of the crop canopy) and background (including the remaining pixels apart from the green vegetation).

**Table 1.** Detailed description of manually labeled real image datasets for wheat and rice crops.

Crop	Camera	GSD (mm/pixel)	Site	Weather	Growth period	Number of images
Rice	Canon EOS Kiss x5	0.1–0.3	Tokyo, Japan	Sunny, cloudy,	Transplanting, tillering, jointing	550
	SONY RX0	0.3–0.5	Jiangsu, China	Sunny, cloudy	Tillering, jointing, heading, filling	400
	^NIKON D7100^	0.3–0.5	Wuhan, China	Shade	Jointing, flowering	100
Wheat	Garden Watch Cam	0.3–0.4	Tokyo. Japan	Sunny, cloudy, rainy	Tillering	300
	SONY RX0 II	0.3–0.5	Montpellier, France	Sunny, cloud	Tillering, standing	200
	Canon 5D Mark II	0.3–0.5	Zurich, Switzerland	Sunny, cloudy	Emergence, tillering	90
	SONY RX0	0.4–0.6	Jiangsu, China	Sunny, cloudy	Tillering, standing, jointing, Filling	300

### Image simulation using D3P

**Fig. 1.**
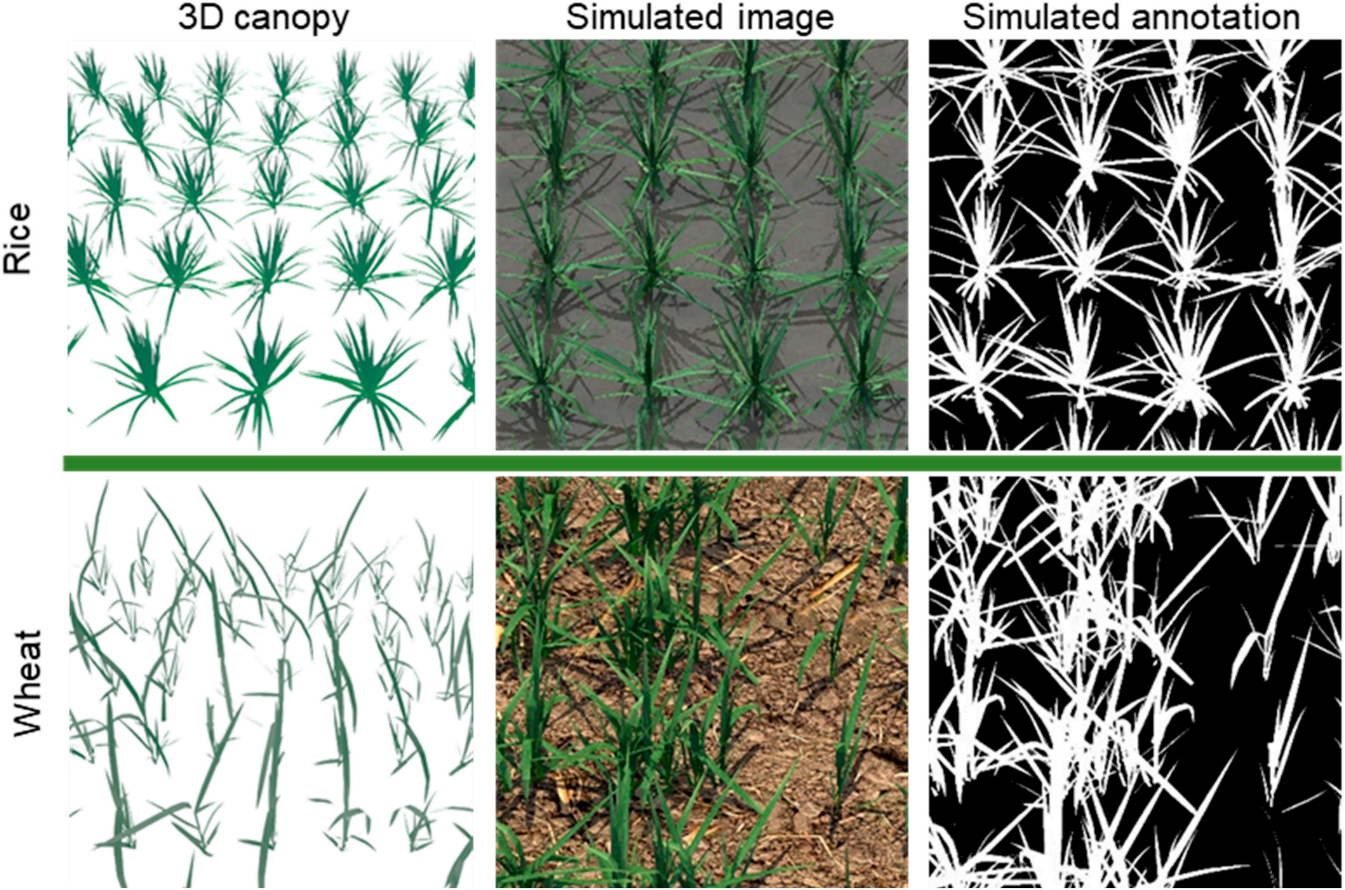
Simulated images and corresponding labels of rice and wheat generated using D3P. From left to right are the original 3D canopy generated by D3P, the simulated RGB image captured after rendering the background and lighting using POV-Ray, and the corresponding labels.

We generated simulated images for wheat and rice crops and the corresponding annotations automatically for semantic segmentation using the D3P (Fig. 1). D3P developed in our previous work consists of 2 parts: a functional structural plant model to generate 3D virtual canopies and a physically based ray tracer to simulate images [25]. For wheat crops, we used SOTA functional structural plant model and ADEL-Wheat models [33,34]. By manipulating the input parameters, D3P allows the simulation of the dynamics of various canopy structures, considering genotypic and environmental impacts. Because rice is morphologically similar to wheat, we used the ADEL-wheat model to simulate 3D virtual rice canopies by modifying the plant arrangement (density and row spacing) as well as the canopy structure, especially the leaf curvature, and inclination.

D3P acquires RGB images from a 3D virtual canopy by mimicking the behavior of a real-world camera. In this study, we set the virtual camera at 1.5 height, 85^o^ field of view, 45^o^ inclined, and 1,024 × 1,024 resolution and sampled 500 times from the parameter space using Latin-hypercube sampling and 4 growth stages (including 227 parameters). With respect to each set of parameters, D3P simultaneously generates both the RGB images and the corresponding labels for semantic segmentation. Using our local desktop computer (11th Gen Intel Core i7-11700@ 2.50GHz, NVIDIA GeForce RTX 3060), we spent <100 h generating the simulated dataset, including 2,000 images and the corresponding annotation for both rice and wheat crops (then cropped to 512 × 512 size with 8,000 images, named the sim dataset hereafter).

### Domain adaptation with CycleGAN

**Fig. 2.**
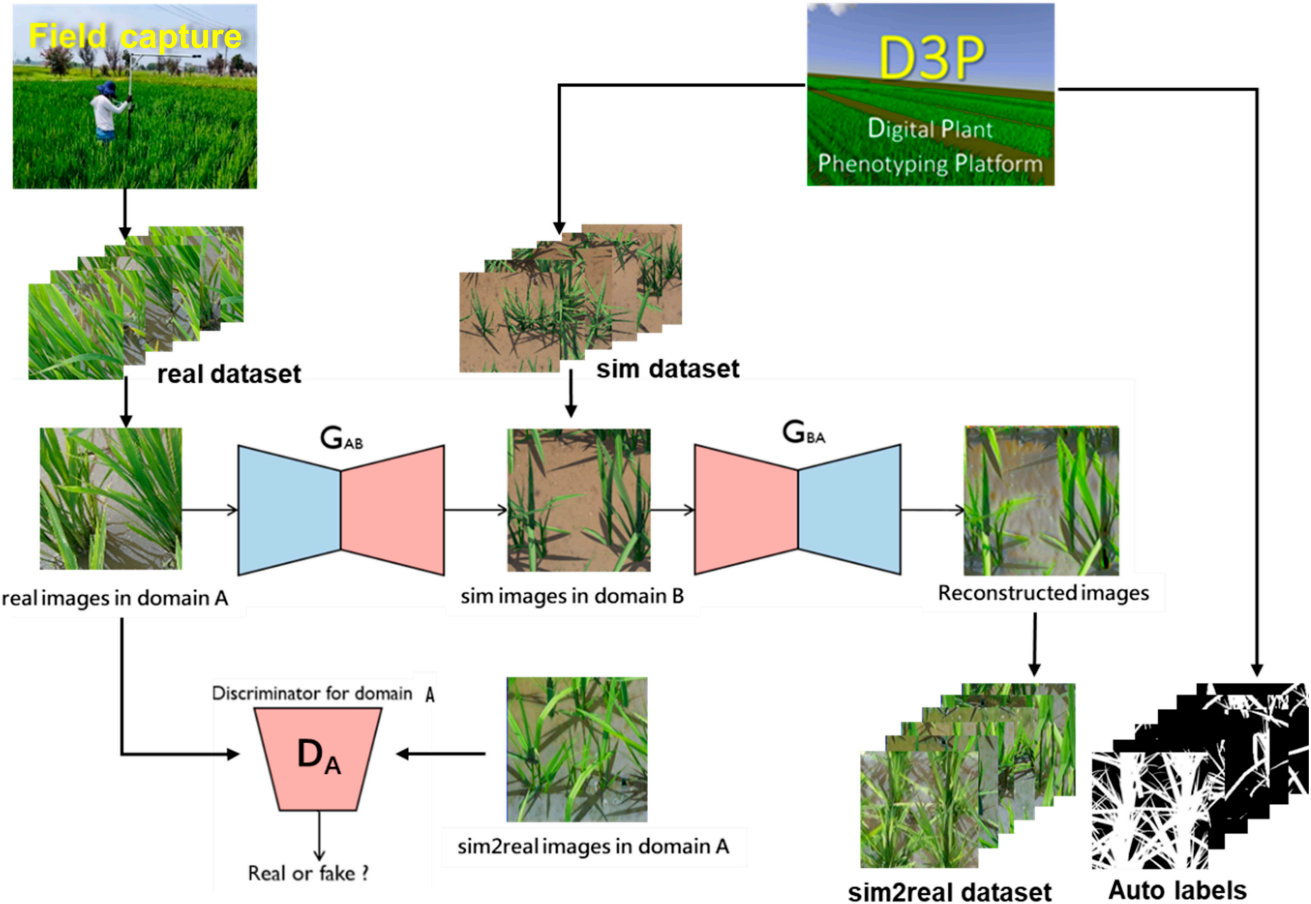
Use of CycleGAN to enhance sim images. G_AB_ and G_BA_ are 2 generators. D_A_ is a discriminator. The real images taken in the field and the sim images generated by D3P are cropped to 512 × 512 size and input to the CycleGAN model for training. The trained G_BA_ weights are used to convert the sim images into sim2real images.

Because there is still a domain gap between the real and sim datasets, we used CycleGAN to close the gap between the 2 feature domains and enhance the realism of the images [30]. CycleGAN simultaneously trains the forward and backward translators between the real and fake domains with a cycle consistency loss that encourages images belonging to the 2 domains to be indistinguishable (Fig. 2). In this study, we trained CycleGAN using 700 images from the real dataset and 700 images from the sim dataset. The computation was conducted on a desktop with an Intel Core i7-11700F CPU processor (16 GB of RAM), a Quadro RTX 8000 card (48 GB video memory), and an operating system with Ubuntu, using Python 3.7 programming language to implement the construction. The hyperparameters were set as an Adam optimizer with a momentum factor of 0.5, batch size of 1, and an initial learning rate of 0.0005. The trained network was then applied to translate the simulated images so that we could create a simulation-to-realism dataset (sim2real dataset) with 8,000 images. To evaluate the similarity between the synthetic and real images before and after domain adaptation, we calculated the Euclidean metric to quantify the similarity between the images [35].

### Model training and assessment

1. Candidate semantic segmentation models

The following 3 candidate models were used: U-Net [36], DeepLabV3+ [37], and SegFormer [38]. These were selected because the 3 models have distinct network structures and all have achieved SOTA. (a) U-Net: It is developed from fully convolutional networks [39] and named by its U-shaped symmetric structure, where the encoder acts as a convolution for feature extraction and the decoder is up-sampled to recover the spatial structure of the image. Because of the simplicity of its structure, it can provide good segmentation accuracy even with limited training samples [40–42]. (b) DeeplabV3+: Depth-wise separable convolution was applied to both the atrous spatial pyramid pooling and decoder modules. The new decoder structure allows the boundaries of the target to be captured by gradually recovering the spatial information clearly [43]. Hence, SOTA was applied to several natural image datasets (e.g., VOC2012). (c) SegFormer: It redesigns a hierarchically structured transformer encoder and lightweight multilayer perceptron decoder. It sets a novel SOTA in terms of efficiency, accuracy, and robustness in several semantic segmentation datasets (e.g., ADE20K, Cityscapes, and Coco Stuff) [44].

2. Model training

All 3 candidate models were trained for both wheat and rice crops using the real, sim, and sim2real datasets. Subsequently, their performance was tested on several independent test datasets with real images (Table 2). To implement the construction, we deployed the semantic segmentation frameworks on a computer platform with an Intel Core i7-11700F CPU processor (16 GB of RAM), GeForce TITAN V GPU card (12 GB of video memory), and an operating system with Windows 10, using the Python 3.7 programming language to implement the construction. Following official advice, we trained U-Net and DeeplabV3+ with EfficientNet-B3 and ResNet-101, respectively, as the backbones. SegFormer uses a default vision transformer as the backbone. The initial weights were initialized using weights pretrained using ImageNet-1 k. The hyperparameters were set as Adam optimizer with a momentum factor of 0.9 and the decay was 0.01. The learning rate was set to the initial value of 0.0001. The number of learning epochs was set to 100, and the batch size was 4. Before the training was completed, the deep learning network automatically saved the model with the lowest loss.

**Table 2.** Dataset composition for 9 training strategies (3 training datasets and 3 semantic segmentation models). The numbers indicate the number of images used.

Crop	Dataset	Training and validation			Testing
		U-Net	DeepLabV3+	SegFormer	
Rice	real	750	750	750	real: 300
	sim	8,000	8,000	8,000	
	sim2real	8,000	8,000	8,000	
Wheat	real	600	600	600	real: 290
	sim	8,000	8,000	8,000	
	sim2real	8,000	8,000	8,000	

3. Assessment metrics at the pixel and image scales

First, the domain gap between the real and synthetic datasets and the performance of the candidate models were evaluated at the pixel scale using the Euclidean metric, Accuracy, and F1-score. We further inspected the confusion matrix to compare the classification accuracies of the pixels. Second, the GF was extracted for each segmented image, corresponding to the number of green pixels divided by the total number of pixels. The GF estimation results obtained from the semantic segmentation models were compared with those from the manual labels and used to calculate *R*^2^ and root-mean-square error (RMSE). The formulas for these metrics are listed in Table 3.

**Table 3.** Metrics used to evaluate the performances of the models.

Scale	Metrics	Name	Formula
Pixel scale	Euclidean metric	Euc	$\sqrt{\sum {\left(x_{i}-y_{i}\right)}^{2}}$ (*x_i_*,*y_i_* is the DN value of the corresponding pixel of the image)
	True positive	TP	Proportion of pixels well predicted in green crop class
	True negative	TN	Proportion of pixels well predicted in background class
	False positive	FP	Proportion of pixels wrongly predicted in green crop class (confusion)
	False negative	FN	Proportion of pixels wrongly predicted in background class (missing pixels)
	Accuracy	Acc	$\frac{\mathrm{TP}+\mathrm{TN}}{\mathrm{TP}+\mathrm{TN}+\mathrm{FP}+\mathrm{FN}}$
	F1-score	F1	$\frac{2\times \mathrm{TP}}{2\times \mathrm{TP}+\mathrm{FP}+\mathrm{FN}}$
Image scale	Coefficient of determination	*_R_* ^2^	$1-\frac{\sum {\left(f_{i}-\bar{y}\right)}^{2}}{\sum {\left(y_{i}-\bar{y}\right)}^{2}}$
	RMSE	RMSE	$\sqrt{\frac{\sum {\left(f_{i}-y_{i}\right)}^{2}}{n}}$ (*f_i_* is the predicted value, *y_i_* is the measured value, $\bar{y}$ is the mean value of the measured value, and *n* is the sample size)

4. Uncertainty analysis

Some studies have reported that the accuracy of deep learning tasks becomes uncertain if pixels appear in images that were too dark or too bright [7,45]. In addition, the appearance of unlabeled classes (e.g., senescent plant elements, which were defaulted as background in this study) can also result in incorrect classification by the model [14,17]. Therefore, we selected images from the test dataset and created 2 specialized datasets for brightness inhomogeneity and senescent leaves, separately. We then used error pixel visualization to observe segmentation errors in the image. To further discuss the uncertainties caused by these 2 factors, we calculated 2 new values for each pixel in all the test images. We used the L channel value extracted from the CIE LAB color space [46] to reflect the inhomogeneity of illumination. The Excess Green value (ExG = 2G − R − B) was calculated to reflect the greenness. To count the L and ExG values per pixel of all images in the rice and wheat images datasets, the distributions of the L and ExG values for all TP, TN, FP, and FN pixels were systematically evaluated using kernel density estimation.

## Results

### Domain gap after domain adaption

**Fig. 3.**
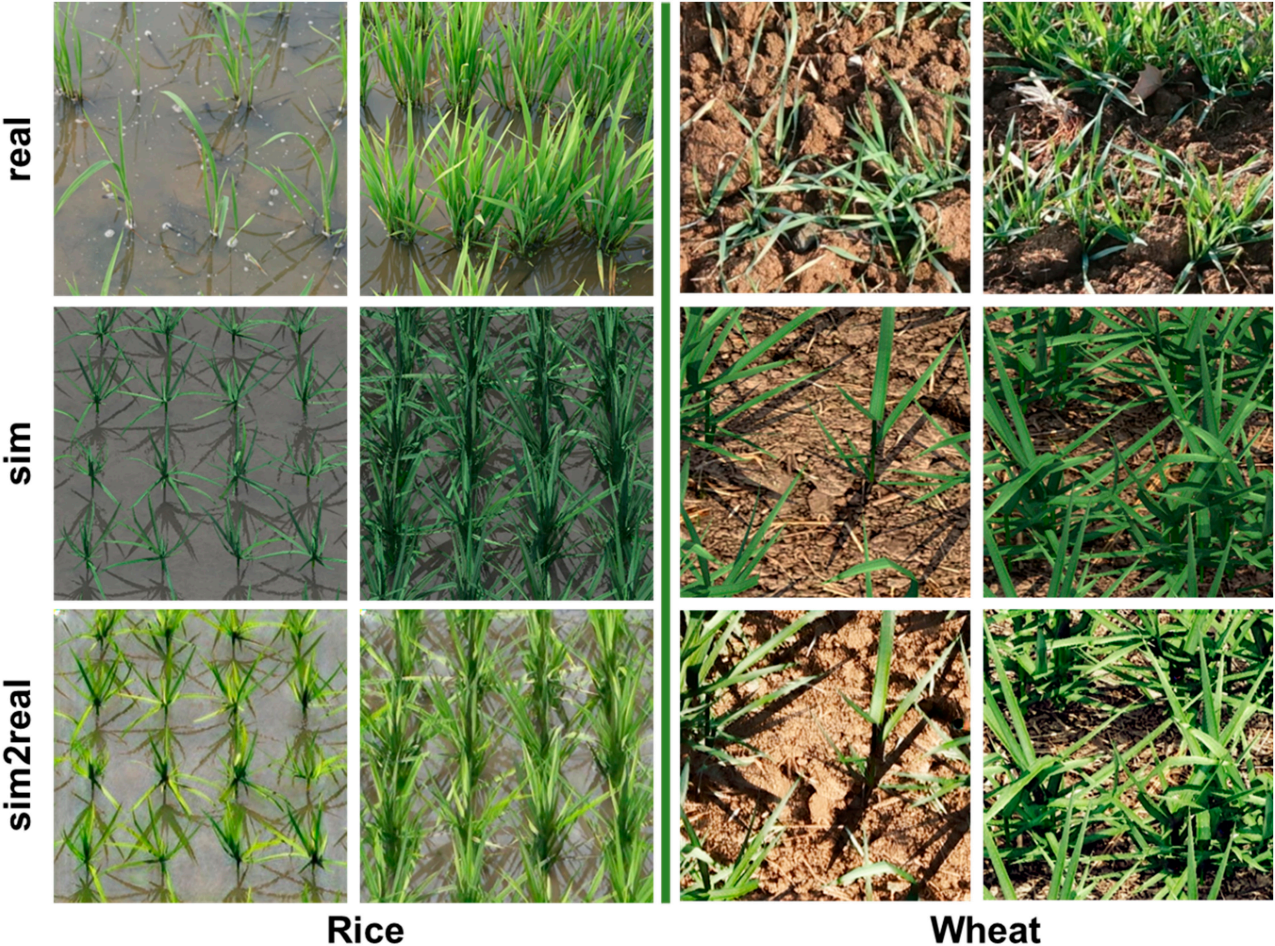
Illustrations of real images, sim images, and sim2real images of rice and wheat crops.

After domain adaptation using CycleGAN, sim2real images appeared more realistic than sim images for both rice and wheat crops. Not only the colors and textures of the plants were closer to reality, but the soil background was also rendered fuller (Fig. 3). Furthermore, Table 4 shows that the Euclidean distance between the sim2real and real images (rice: Euc = 0.301, wheat: Euc = 0.375) was smaller than that between the sim and real images (rice: Euc = 0.343, wheat: Euc = 0.437). The assessment, both visually and quantitatively, proved that CycleGAN can effectively reduce the domain gap between the simulated and real images.

**Table 4.**
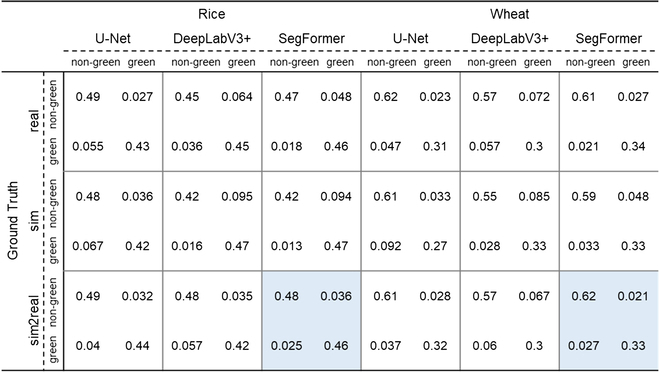
Euclidean distance before and after domain adaptation and pixel scale segmentation performance of 9 training strategies in the wheat and rice test dataset. Blue box: best result.

### Semantic segmentation performance at pixel scale

**Fig. 4.**
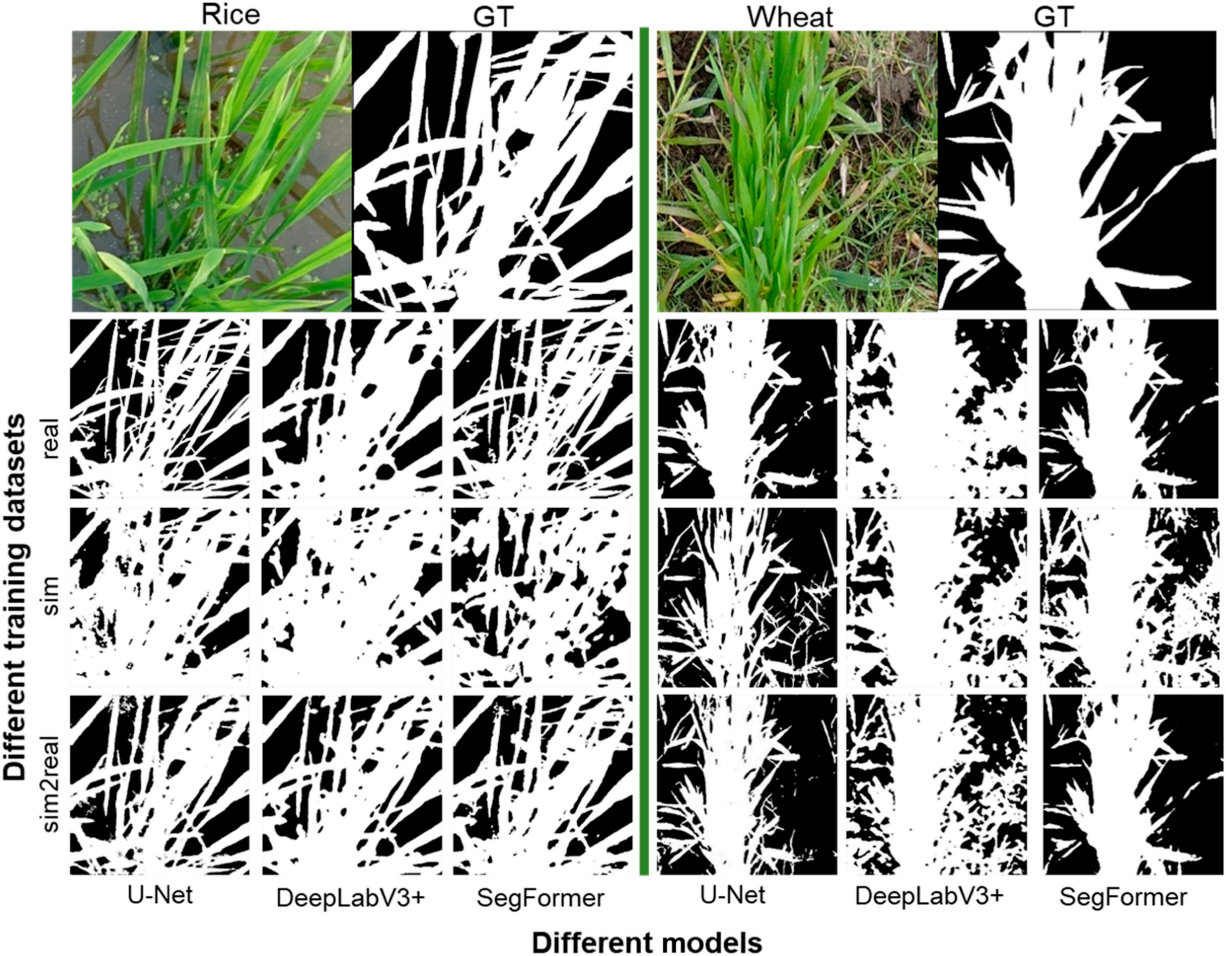
Visualization of 9 training strategies tested on real rice and wheat images. GT, ground truth.

U-Net and SegFormer provided similar segmentation results for both rice and wheat crops, whereas DeepLabV3+ performed obviously worse than the other 3 segmentation models (Table 4). Figure 4 shows that DeepLabV3+ misclassifies weeds as green pixels in wheat crops. SegFormer achieved the highest F1-score and Accuracy compared with the other models. Among the 3 datasets, all models obtained the highest F1-score and Accuracy using the sim2real dataset. However, we found that with SegFormer, the Accuracy using the sim2real dataset was only slightly better than that when using the real dataset. Moreover, the 3 models performed the worst in the sim dataset. These trends were similar for wheat and rice crops. Overall, training SegFormer with the sim2real dataset outperformed all the other approaches. In terms of the F1-score, the segmentation results were slightly better for rice, whereas the opposite was true for the Accuracy (rice: F1 = 0.937, Acc = 0.940; wheat: F1 = 0.935, Acc = 0.952).

We further inspected the performances of the 9 strategies using a confusion matrix (Table 5). Training SegFormer with the sim2real dataset achieved the highest sum of TP and TN for both rice and wheat crops (rice: TP = 0.46, TN = 0.48; wheat: TP = 0.33, TN = 0.62). We then inspected the FP and FN. For wheat, the misclassified pixels had approximately the same probability as FP (0.021) and FN (0.027), for rice, the misclassified pixels were more likely to be FP (0.036) than FN (0.025) pixels. This indicated that the background pixels in the rice images were more likely to be classified as green crop pixels than those in the wheat images. In contrast, there was the same possibility of misclassification of the green crop pixels as the background.

**Table 5.**
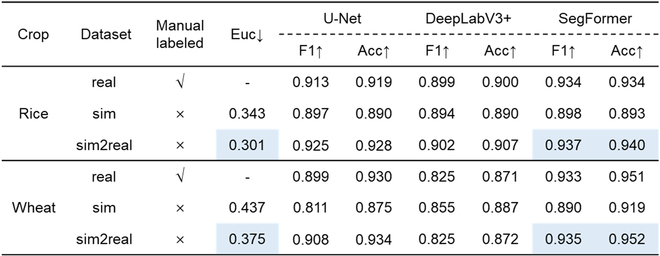
Pixel scale evaluation of 9 training strategies with a confusion matrix for wheat and rice crops. Blue box: the best result. TP: green classified as green; TN: nongreen classified as nongreen; FP: nongreen misclassified as green; FN: green misclassified as nongreen.

### GF estimation performance

**Fig. 5.**
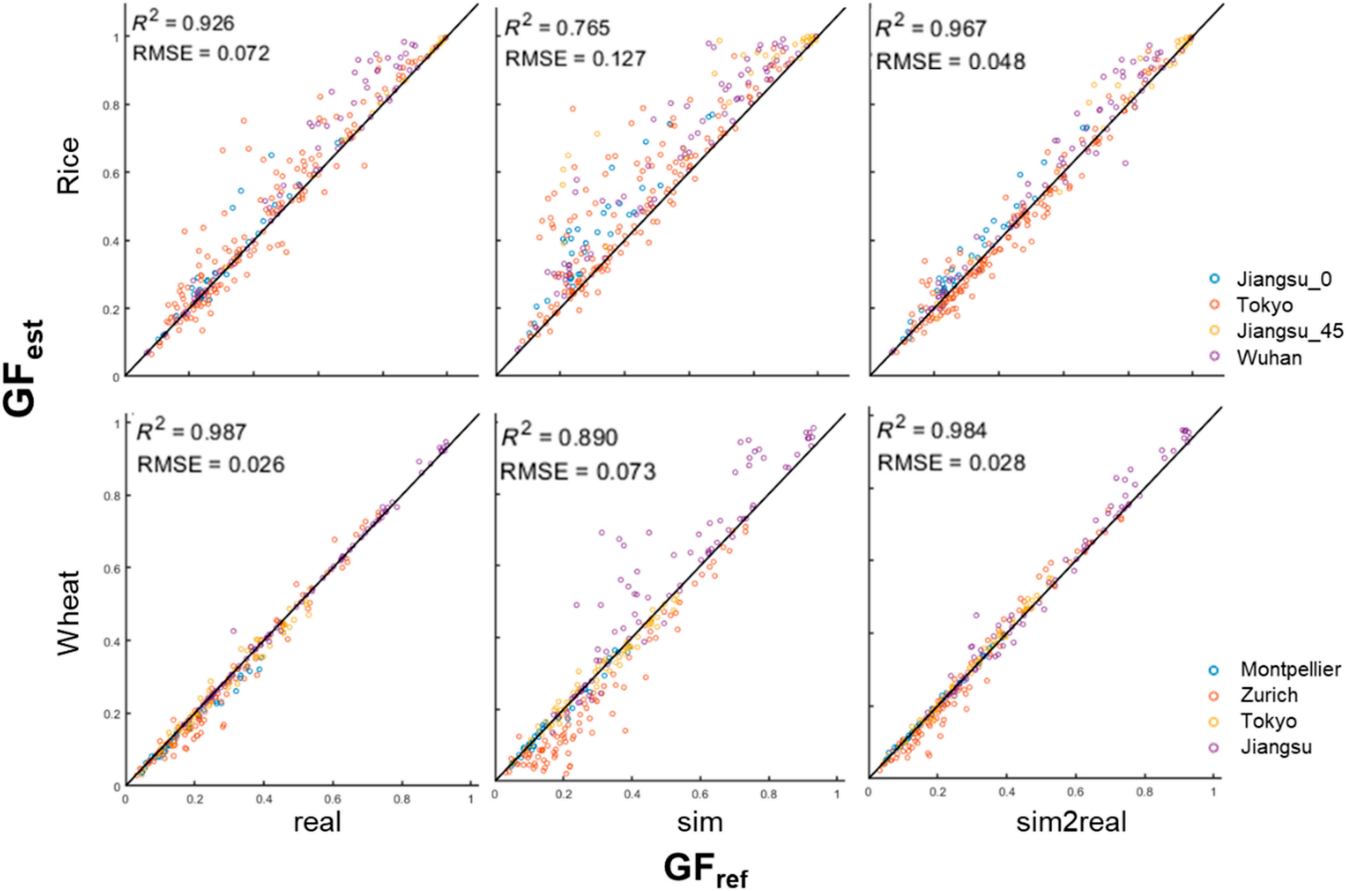
Performance of GF estimation with Segformer model trained over 3 datasets. The performance was tested on real images of rice and wheat collected from different sites and varied configurations. Jiangsu_0 and Jiangsu_45 represent images taken from Jiangsu with a camera inclined at zenith angle 0° and 45°, respectively.

The GF estimation performances of the 9 training strategies for both wheat and rice crops are presented in Table 6. For wheat crops, the GF estimation result achieved by training SegFormer with the sim2real dataset (*R*^2^ = 0.984, RMSE = 0.027) was very close to that obtained by training SegFormer with the real dataset (*R*^2^ = 0.987, RMSE = 0.026). However, for rice crops, the GF estimation achieved a better result by training SegFormer with the sim2real dataset (*R*^2^ = 0.967, RMSE = 0.048) than with the real dataset (*R*^2^ = 0.926, RMSE = 0.072). Importantly, using SegFormer training with the sim2real dataset, the GF estimation result was performed consistently among the test datasets, whether from different sites or different observation inclinations (Fig. 5).

**Table 6.**
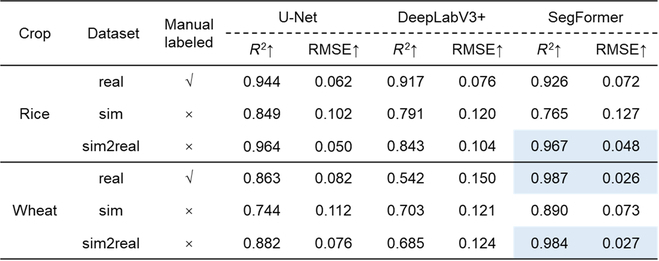
GF estimation performance of the 9 training strategies for wheat and rice crops. Blue box: the best result.

### Dynamics of GF

**Fig. 6.**
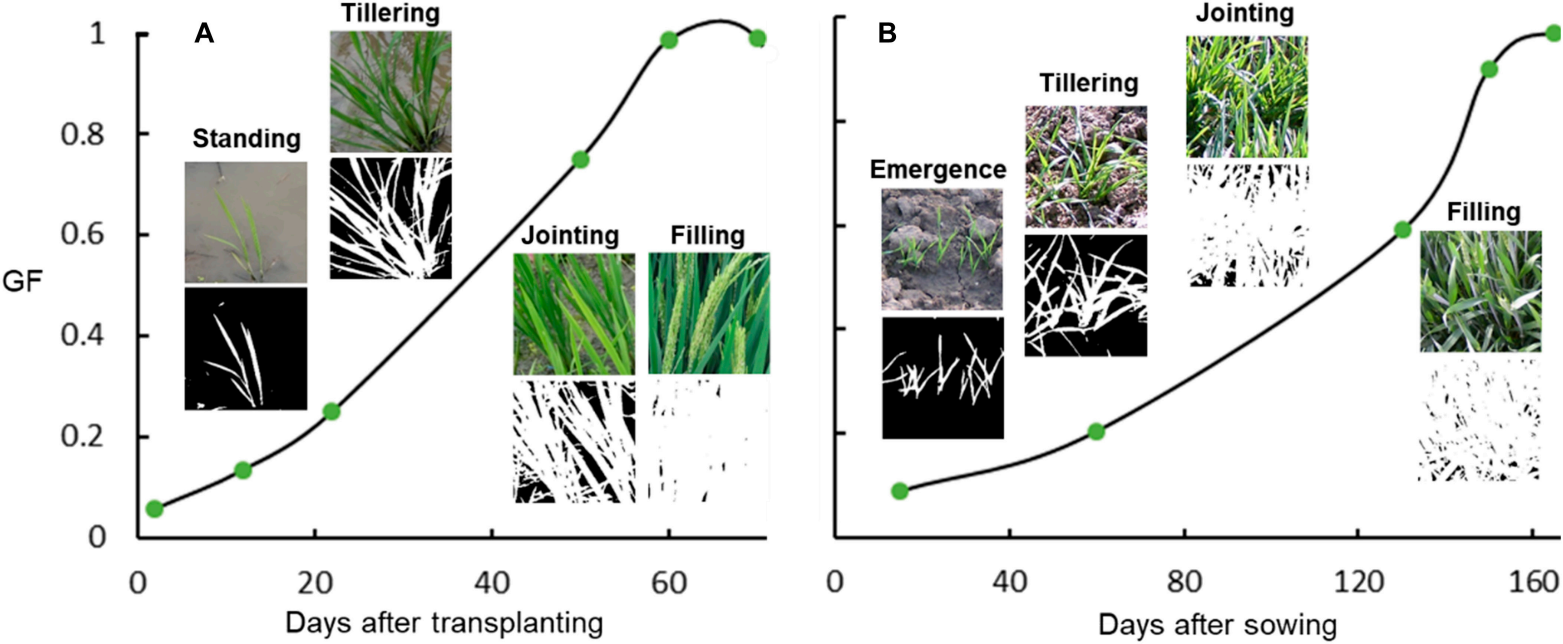
Dynamics of GF generated by images segmented using the optimal strategy (sim2real + SegFormer): (A) Rice and (B) wheat.

Following the assessment, the best approach, SegFormer, trained with the sim2real dataset, was used to segment the time-series images of rice and wheat. Here, we demonstrated the GF dynamics of 1 rice cultivar between tillering and flowering and 1 wheat cultivar between emergence and flowering (Fig. 6). Overall, the GF dynamic curve was smooth, indicating the reality of the GF estimation.

### Uncertainty of segmentation

**Fig. 7.**
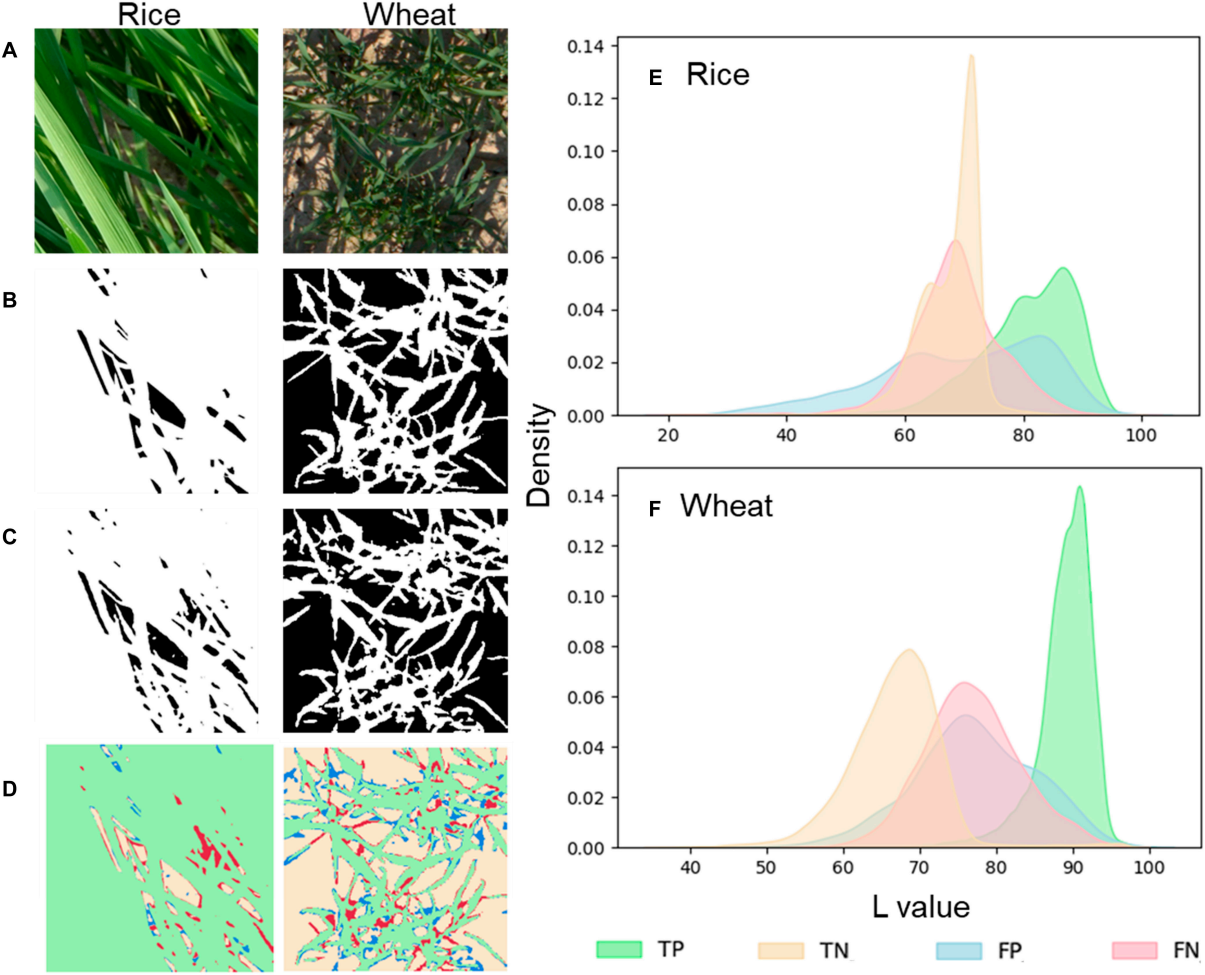
Segmentation error caused by brightness inhomogeneity in the same image. (A) RGB image, (B) GT mask, (C) segmentation mask, (D) error pixel visualization, (E) rice kernel density estimation, and (F) wheat kernel density estimation. (D) to (F) shared the same legend. TP: green crop pixels correctly classified under various lighting conditions. TN: background pixels correctly classified; FP: background pixels misclassified as green crop pixels; FN: green crop pixels misclassified as background pixels. (For interpretation of the references to color in this figure legend, the reader is referred to the web version of this article.)

For the best approach, SegFormer trained with the sim2real dataset, we further explored the critical impact factors for the GF estimation uncertainty. Two main factors were considered including nonuniform brightness within the image and the appearance of senescent leaves. The deep layer of the canopy was shadowed by the top layer of the canopy. This caused nonuniformity of brightness among pixels in the same image, which can be characterized by the L channel of the CIE LAB color space. Figure 7E and F clearly shows that the green crop pixels were much brighter than the background pixels. For rice crops, the category of the misclassified pixels was controlled by their brightness. When they were in a shadow with a relatively low L value, they were more likely to be misclassified as background, corresponding to the FN. Likewise, when they were in the bright region with a relatively high L value, they were more likely to be misclassified as green pixels, corresponding to FP (Fig. 7D and E). However, for wheat crops, misclassified pixels were not directly linked to the brightness. Specifically, brighter background pixels and darker green crop pixels can be misclassified, corresponding to FP and FN, respectively (Fig. 7D and F).

**Fig. 8.**
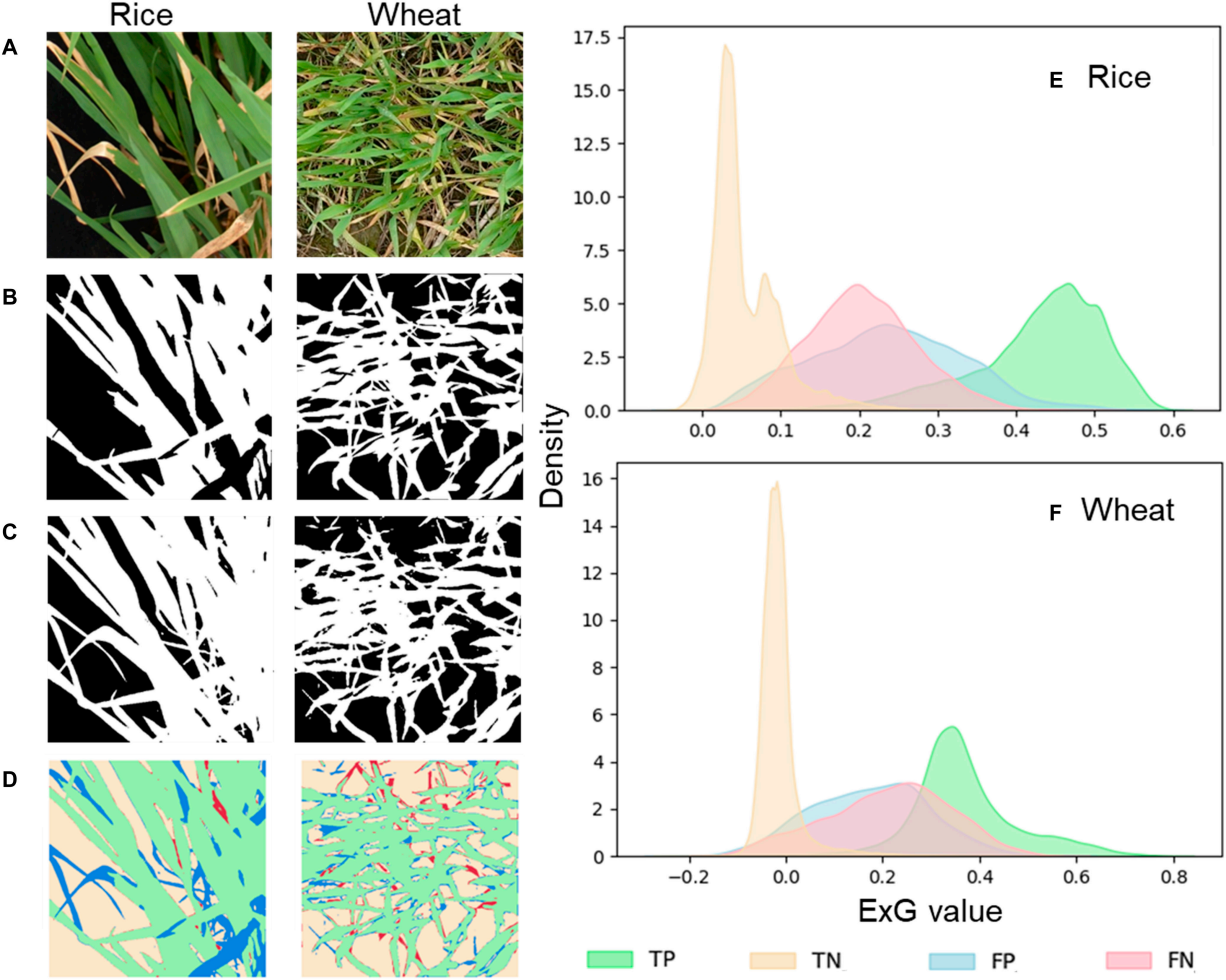
Segmentation errors caused by senescent leaves. (A) RGB image, (B) GT mask, (C) segmentation mask, (D) error pixel visualization, (E) rice kernel density estimation, and (F) wheat kernel density estimation. (D) to (F) shared the same legend. TP: green crop pixels correctly classified; TN: background pixels (contained senescent crop pixels) correctly classified; FP: background pixels (contained senescent crop pixels) misclassified as green crop pixels; FN: green crop pixels misclassified as background pixels. (For interpretation of the references to color in this figure legend, the reader is referred to the web version of this article.)

For rice and wheat crops, when the leaves grow to age, they start to become senescent, with an appearance that is not green enough. The greenness of a pixel can be characterized using the ExG value. Figure 8E and F shows that the green crop pixels and correctly classified background were very contrasting in terms of ExG. As expected, misclassification occurred mainly when the ExG value of the pixels was not close to either the ExG value of the green leaves or background. When comparing the segmentation performance of the rice and wheat crops, we found that the senescent leaves of the rice images were more likely to be misclassified as green leaves (Fig. 8D).

## Discussion

The green crop semantic segmentation paradigm that we developed is called self-supervised in this study because it does not need any human labels to supervise the training. Specifically, D3P can automatically generate many simulated images with highly accurate mask labels, thus establishing the sim dataset. Subsequently, the reality gap between the sim and real datasets can be minimized using the domain adaptation technique to generate a sim2real dataset that can be used to train deep learning models. As mentioned in Introduction, it is difficult to prepare large crop image datasets with high-precision labels, especially segmentation mask labels. To prepare the labels of real dataset, it took 6 people a total of 1,000 h to annotate 1,900 images. Our self-supervised segmentation approach would save all the time spent on image annotation. Furthermore, the model performance benefit from the diverse sim2real dataset with the high-precision labels.

A self-supervised plant phenotyping pipeline that focuses on the leaf counting of wheat crops was developed in our recent study [29]. In this study, we successfully extended this strategy to semantic segmentation of ground-based rice and wheat RGB images for the first time. These 2 works used similar self-supervised approach that requires no human labels to supervise the model training. However, they put focus on 2 distinct tasks, object detection and semantic segmentation, respectively. The results achieved here would further prove the validity and generality of our self-supervised approach for the development of phenotyping algorithm.

Whether rice or wheat crops that with very contrasting field backgrounds, SegFormer trained with the sim2real dataset outperformed all other approaches at both pixel and image scales (Tables 4 and 6). The GF estimation performance was very consistent among datasets taken from different sites with different camera configurations for rice and wheat, with RMSE = 0.048 and RMSE = 0.028, respectively (Fig. 5). Comparing previous work of outdoor rice and wheat image segmentation with shallow methods [7,12,47], even the recently published model SegVeg [17], our proposed self-supervised method performs SOTA in terms of accuracy and dataset production. This demonstrates that self-supervised learning can replace the traditional methods for wheat and rice image annotation and analysis. The developed model allowed us to monitor the dynamics of GF for rice and wheat crops using ground-based RGB images. This would meet breeders' requirements to characterize the process of crop growth and development and consequently guide cultivar selection.

It is worth noting that SegFormer trained with the sim2real dataset achieved an accuracy comparable to that of SegFormer trained with a real dataset, indicating that the sim2real dataset is as efficient as the real dataset for model training. We believe that the huge advantages in quantity and quality ultimately make the sim2real dataset the best performance. Specifically, the sim2real dataset containing 8,000 perfectly labeled images is far larger than the size of the real dataset (<800 images). The number of images and the high quality of the mask labels in the sim2real dataset ensure that the deep learning model can accurately extract features from a wider domain.

The crops in our simulated images have realistic and various 3D structures, which is mainly attributed to the D3P simulations. First, a 3D plant model is incorporated into the D3P, which allows the simulation of the dynamics of the canopy structure based on ecophysiological mechanisms. This ensures the realism of the canopy structure and provides the feasibility of generating various canopy structures by manipulating model parameters. Furthermore, the images are rendered using a semiphysically based software, POV-Ray. We can simulate the images under various illumination conditions and simultaneously produce corresponding mask labels. All these factors ensure that the domain of the simulated image is not too far from realism (Fig. 1). Even so, the reality of the simulated images would continuously benefit from the improvements in the 3D plant model. Ideally, if a 3D model is sufficiently realistic, there will be no reality gap. We could then directly use the simulated dataset to train the model without domain adaptation.

Although the simulated images appear visually similar to the real images, a large domain gap still exists between the simulated images generated by the D3P and the real images captured in the field. The overall style of the simulated image still needs to be adapted, including the leaf texture, background, and light distribution (Fig. 3). It is impossible to obtain satisfactory segmentation results when training the model with the sim dataset (Tables 4 and 6). Because CycleGAN does not require paired images for model training and respects the boundaries of objects in the image, it was used to transfer the simulated images to the style of real images. CycleGAN demonstrated efficacy in both rice and wheat crops with very different backgrounds (Table 4). However, our experience indicates that training CycleGAN is not always easy to converge, especially when the image resolution is coarse with a complex background. Nevertheless, it is possible to replace CycleGAN with other SOTA domain adaptation algorithms [48].

Among the 3 candidate semantic segmentation models, SegFormer outperformed DeepLabV3+ and U-Net regardless of the training dataset (Table 4 and Fig. 4). SegFormer trained with the sim2real dataset can precisely segment images with green weeds, which is often considered a major challenge for segmentation algorithms [49]. Unlike U-Net and DeepLabV3+, based on a convolutional neural network, SegFormer has a unique transformer mechanism [38]. Thus, SegFormer has a larger effective receptive field for potentially learning more representative global features. This contributes to distinguishing the green crop pixels from the background. In addition, DeepLabV3+ performed worse than U-Net in this study. This is different from most of the existing reports, probably because of the inconsistent image sizes among the model training datasets. DeepLabV3+, based on atrous spatial pyramid pooling, contains convolution kernels of different sizes. Stacking these convolution kernels helps DeepLabV3+ better capture global features on large-sized images (e.g., road and indoor panoramas). Conversely, the image size was small in our study (256 × 256 and 512 × 512), which makes it difficult for the convolution kernels to extract small features.

The overall performance of the segmentation model was slightly degraded when senescent leaves were present in the image (Fig. 5). In addition, our approach is limited to the stage before the grain filling. This is because D3P is not capable of simulating the senescent processes well, particularly after grain filling. There were no senescent leaves in the dataset even after domain adaptation. Furthermore, when comparing the performance of rice and wheat images, the confusion matrix (Table 5) and uncertainty analysis demonstrated that senescent leaves in rice images were more likely to be misclassified than those in wheat images (Fig. 8D). This is probably because, when simulating rice images, we simply considered the background of the paddy rice field as a flat water surface that appears light gray with a specular effect. No yellow elements were observed in the training dataset. In contrast, the soil background of the wheat field is yellow, which may help to teach the segmentation model to classify the senescent leaves. In the future, we will improve the ecophysiological processes incorporated into the D3P, consequently increasing the realism of simulated images involving senescent processes for both wheat and rice crops. Furthermore, applying more sophisticated domain adaption models would better consider background diversity and illumination changes in the simulated image. These improvements will contribute to the capacity of the segmentation model and the accuracy of GF estimation over the entire growth cycle.

## Data Availability

The datasets could be given upon reasonable request from the corresponding author.
